# Improving Student Attitudes Toward Palliative Care

**DOI:** 10.1177/1049909120931800

**Published:** 2020-06-09

**Authors:** Amandeep Pahal, Mohini Bhagalia, Bianca Goonewardena

**Affiliations:** 1 Medical School, St George’s, University of London, United Kingdom; 2 Medical School, University of Leicester, London, United Kingdom

We read with great interest the article titled “University Student’s Attitude Toward Palliative Care.”^1^ With an aging population growing at an exponential rate, palliative care is becoming a fundamental component of our health care systems. Intriguingly, studies have shown that health care professionals lack the skills and knowledge in dealing with end-of-life care.^2,3^ Many students fail to understand the objectives of palliative care and feel underprepared when faced with end-of-life situations.^4^ It is crucial that all physicians and medical trainees possess a positive attitude toward the specialty.

As United Kingdom medical students, we appreciate that attitudes toward palliative care differ within our respective universities. This is a result of the varied clinical exposure to the specialty. During preclinical years, most medical schools focus on the definitive management of conditions and students are left unfamiliar with the process of dying. Contrarily, St George’s preclinical medical students are offered a series of lectures on the process of dying. Discussing the components of a good death encourages students to understand the importance of palliative care in improving quality of life for patients. Students were given the opportunity to discuss the negative attitudes of physicians toward the specialty. These lectures are a useful stepping stone in preparing medical students for the palliative care rotation in clinical years. Furthermore, students receive clinical communication sessions on handling difficult discussions. However, the breaking bad news workshops are taught in final year. We believe that earlier incorporation of such workshops into the curriculum would allow students to implement these skills in palliative care placements in fourth year.

During palliative care rotations, students are randomly allocated to different hospices; some offering more opportunities than others. At the hospice, we were encouraged to participate in a wide range of activities to gain further insight into end-of-life care. The length of hospice placements varies throughout different universities, creating discrepancies in knowledge and insight within palliative care. We propose that a hospice rotation, of at least 1 week in duration, is implemented as a requirement across medical schools nationwide. As demonstrated by Miltiades, there is a positive correlation between experience and positive attitudes.^1^ This supports the argument that experience and exposure to a hospice environment is fundamental for medical students. From our own experiences, we have observed that those with longer hospice placements are more comfortable when dealing with discussions around death and dying.

Participating in Multidisciplinary team meetings enabled us to appreciate the role of different health care professionals in palliative care. We learnt the importance of taking a holistic approach to meet patients’ needs, considering not only medical aspects of care but also social, psychological, and spiritual. Observing physicians in other specialties suggested that this holistic approach is not always adapted, as many physicians are untrained in taking a spiritual history. This remains a crucial part of end-of-life care as evidence demonstrates there is a strong link between faith and the ability to cope with an illness.^5^ It is essential that alongside medical students, other health care students receive early end-of-life teaching.

Hospice ward rounds are a fundamental teaching resource. Students became familiar with the various patients at different stages in end-of-life care. While some patients required symptom control and optimal comfort in their last few days, others attended as outpatients participating in activities for mental well-being. Therefore, it is essential that medical professionals can recognize at what point one may benefit from palliative care. This timely decision can improve an individual’s quality of life and prevent further suffering. For instance, patients with cancer will inevitably require palliation due to the demarcation between the curative and palliative phase. However, for patients with chronic long-term conditions such as chronic obstructive pulmonary disease, there is ambiguity in the process of involving palliative care.^6^ Research shows that physicians are unable to promptly recognize the need for palliative care. This is due to their inexperience and the lack of resources provided during medical school and residency programs.^7^ Consequently, patients suffer late into their disease trajectory, compromising quality of life and decision-making capacity.

Leicester Medical School organizes a 6-week community-based rotation. Students gain an insight into palliative care in numerous settings, appreciating the importance of transferable skills suited to different environments. Students have the scarce opportunity of completing home visits. We believe these visits significantly improved our attitudes on end-of-life care. Allo et al demonstrated that 95% of physicians agree that home visits were an effective teaching tool.^8^ The lack of training in home-based palliative care is a major hurdle in the implementation of palliative care in the community.^8^ This study states that for many, the preferred place of death is at home.^9^ For this reason, it is crucial that physicians provide a high standard of home-based care. The community-based model implemented in Steinhauser’s study ought to be incorporated into medical school curricula, so future doctors are well equipped for home-based care.^8^ The community-based model will ensure medical students will familiarize oneself with prescribing, managing symptoms, and working in an end-of-life care MDT.

Following hospice rotations, students attended reflective sessions with the purpose of discussing positive and negative viewpoints on palliative care. These sessions are of utmost importance in refining the medical curriculum each year. Clinicians utilize feedback to implement useful teaching methods the following year. Complex palliative care scenarios are also discussed, for those who were unable to receive clinical teaching from senior clinicians during their rotations. These cases provided the students with several methods to cope with end-of-life discussions and medical care.

Considering the current pressures on the National Health Service, we recognize that hospice rotations and home visits can be difficult to organize for mass scales of medical students. Nevertheless, all students will complete a rotation in elderly care; a team that works closely with the hospital palliative care team. Students ought to utilize this opportunity to arrange any shadowing or teaching sessions. The elderly care team may aid students in discovering valuable resources such as “The Gold Standards Framework.”^10^

Due to increasing exposure to palliative care, it is vital that all clinicians possess a positive attitude toward the specialty in order to provide patients with high quality care. Hence, medical education ought to incorporate aspects of palliative care in preclinical years and increase community-based experience during clinical years.
